# Increased plasma mannose binding lectin levels are associated with bronchiolitis obliterans after lung transplantation

**DOI:** 10.1186/1465-9921-13-56

**Published:** 2012-07-04

**Authors:** Steven J Budd, Robert M Aris, Ayorinde A Medaiyese, Stephen L Tilley, Isabel P Neuringer

**Affiliations:** 1 Protochips, Raleigh, NC, USA; 2 Division of Pulmonary and Critical Care Medicine, University of North Carolina, Chapel Hill, NC, USA; 3 Pikeville Medical Cen, Pikeville, KY, USA; 4 Pulmonary and Critical Care Unit, Massachusetts General Hospital, 148 Bulfinch Bldg., 55 Fruit Street, Boston, MA, 02114, USA

**Keywords:** Mannose binding lectin, Lung transplantation, Bronchiolitis obliterans syndrome

## Abstract

**Background:**

Long-term lung allograft survival is limited by bronchiolitis obliterans syndrome (BOS). Mannose binding lectin (MBL) belongs to the innate immune system, participates in complement activation, and may predispose to graft rejection. We investigated mannose binding (MBL) during cold ischemia and in tissue samples from explanted lungs with BOS, and assessed MBL and complement proteins in plasma post-lung transplantation relative to BOS staging.

**Methods:**

MBL was detected by immunohistochemistry lung tissue at the time of cold ischemia and in samples with BOS. MBL was assayed in the peripheral blood of 66 lung transplant patients transplanted between 1990–2007.

**Results:**

MBL localized to vasculature and basement membrane during cold ischemia and BOS. Patients further out post-lung transplant > 5 years (n = 33), had significantly lower levels of MBL in the blood compared to lung transplant patients < 5 years with BOS Op-3 (n = 17), 1738 ± 250 ng/ml vs 3198 ± 370 ng/ml, p = 0.027, and similar levels to lung transplant patients < 5 years with BOS 0 (n = 16), 1738 ± 250 ng/ml vs 1808 ± 345 ng/ml. MBL levels in all BOS 0 (n = 30) vs. all BOS Op-3 (n = 36) were 1378 ± 275 ng/ml vs. 2578 ± 390 ng/ml, p = 0.001, respectively. C3 plasma levels in BOS 0 (n = 30) vs. BOS Op-3 (n = 36) were 101 ± 19.8 mg/ml vs. 114 ± 25.2 mg/ml, p = 0.024, respectively.

**Conclusions:**

MBL localizes within the lung during graft ischemia and BOS, higher levels of plasma MBL are associated with BOS Op-3 and < 5 years post-transplant, and higher level of plasma complement protein C3 was associated with BOS Op-3 clinical status. MBL may serve as a biomarker for poorer outcome post-lung transplantation.

## Background

Lung transplantation for end-stage lung disease is a successful treatment, however, long-term survival is limited by bronchiolitis obliterans (BOS). The 5-year probability of freedom from BOS is 15-37%, and once diagnosed, actuarial survival is 26-43% [1]. Acute rejection predisposes to chronic lung allograft rejection, and although acute rejection is reversible, repeated injury to the lung allograft by alloimmune and non-alloimmune pathways, will promote airway fibrosis. Innate, adaptive, and humoral immunity also compound airway injury through concurrent infection, Th1 cytokines and T cells, and antibody and complement-mediated injury [2-4]. Acute lung allograft rejection while typically identified by infiltrating T cells, may progress to include peribronchial and perivascular infiltrates with B cells and neutrophils, as well as C3d and C4d complement [5-8]. Antibody-mediated rejection is increasingly recognized as a contributing factor in chronic graft injury and bronchiolitis obliterans and is under intense investigation in thoracic transplantation [9].

Mannose binding lectin (MBL), a member of the collectin family (surfactant proteins A and D, mannose binding lectin, MBL) participates in activation of the alternative pathway of complement, thus bridging adaptive and innate immunity [10]. While surfactant proteins A and D may be protective to the lung allograft after lung transplantation, as reduced levels are associated with BOS [11], MBL may be either protective or harmful depending upon infectious or autoimmune risk. Produced by the liver and secreted into the circulation, MBL is a pattern recognition molecule which binds to pathogens via carbohydrate recognition domains which complex with D-mannose, N-acetylglucosamine, and glucose, activating the lectin pathway of complement [12-14]. Upon recognition of appropriate carbohydrate substrates, MBL binding activates MBL-associated serine protease-2 (MASP-2), with subsequent cleavage of C4 and C2, generation of the C3 convertase C4b2a, and complement activation [15-18]. Low serum MBL levels, often due to genetic polymorphisms, have been associated with increased risk of infection, more severe manifestation of cystic fibrosis lung disease, and autoimmunity, while high levels exacerbate inflammatory diseases and diabetic nephropathy [19-28].

Studies examining the role of MBL in solid organ transplantation have focused mostly upon ischemia-reperfusion injury (IRI), finding that MBL binds to IgM during IRI in experimental models [29]. MBL deposition was observed in association with gastrointestinal and renal transplantation-related ischemia [30-32]. The few studies addressing the role of recipient MBL levels upon long-term allograft survival have identified a detrimental effect associated with elevated MBL levels [33,34]. Studies addressing MBL and lung transplantation have yielded conflicting results, finding increased CMV infection and survival with MBL deficiency [35], high MBL levels associated with worse survival [36], yet low levels of MBL within the airways of patients with BOS [37].

We hypothesize that MBL is deposited within injured lung allograft tissue at the time of transplantation as well as BOS, and elevated in the plasma of patients with bronchiolitis obliterans syndrome (BOS). MBL levels in the plasma of lung transplant patients > 5 years post-transplant with BOS 0 (n = 12) and BOS Op-3 (n = 21) were compared to MBL levels in patients < 5 years with BOS 0 (n = 16) and BOS Op-3 (n = 17).

## Methods

Patient population - In compliance with IRB approved protocols, patients were enrolled through routine clinic appointments, with peripheral blood samples collected during the year 2007 and BOS staging and data analysis performed in 2008. All patients older than 18 years of age were eligible except retransplant. Patients comprised a group > 5 years post-transplant (1990–2003), and a recently transplanted group, < 5 years (2003–2007). A total of 60 patients are alive from the post-transplant group transplanted > 5 years ago, of whom 33 patients were enrolled. In the < 5 years post-transplant, 2003–2007, 54 patients were alive and 33 patients were enrolled form this recent transplant cohort. Patients were staged for BOS according to ISHLT criteria. Characteristics of the patient groups are demonstrated in Table 1.

**Table 1:** Demographics of patients > 5 years post-transplant, compared to patients < 5 years post-transplant

	**< 5 BOS 0**	**< 5 BOS Op-3**	**> 5 BOS 0**	**> 5 BOS Op-3**
n	16	17	12	21
Average age at transplant (all diagnosis groups)	37.9	36.7	36.0	33.5
Average age at transplant (CF)	33.6	25	33.5	31.4
Male/Female	8/8	12/5	4/8	10/11
Diagnosis				
CF	13	11	8	16
Bronchiectasis or PCD	0	0	1	2
COPD or alpha1	1	3	1	2
IPF or sarcoid	2	2	1	1
PH	0	1	1	0
Single lung	0	1	1	2
Double lung	16	16	11	19

Immunohistochemistry – Surgical biopsy tissue samples were analyzed for MBL in 1 ) lung allograft tissue from patients at the time of lung implantation, paralleling cold ischemia, 2) explanted lungs with bronchiolitis obliterans, and 3) lung tissue with diffuse alveolar damage from non-lung transplant patients, serving as a positive control. Tissue was obtained in accordance with IRB protocols in place for sampling of lung tissue during cold ischemia, bronchiolitis obliterans in explanted lungs, and diffuse alveolar damage, granted through UNC Department of Pathology and UNC Cystic Fibrosis Center. Human lung tissue was fixed in formaldehyde and embedded in paraffin. Antigen retrieval was performed. Endogenous peroxidases were blocked. Anti-mannose-binding lectin (human), clone HYB 131–01 (The Antibody Shop, Assay Designs, Ann Arbor, MI) was applied at a dilution of 1:200. Secondary biotinylated donkey-anti-mouse antibody was applied at 1:4000 (Jackson Immunoresearch, West Grove, PA). Streptavidin-horse-radish peroxidase was applied, followed by diaminobenzidine (DAB) detection and methylene green counterstain. Semi-quantitative morphometric analyses of percent vasculature, airway basement membranes, and alveolar-capillary junctions staining positive per slide were performed by a blinded reviewer.

MBL, C3, and C4 in plasma – Mannose binding lectin, C3, and C4 levels were determined in the peripheral blood of all 66 lung transplant patients studied. Peripheral blood was obtained during routine clinic appointments from 33 post transplant patients transplanted within the past 5 years, and 33 post transplant patients transplanted > 5 years. Patients were staged for BOS according to ISHLT BOS Staging criteria. Blood was assayed for MBL (Mayo Medical Labs, Rochester, MN, range 5 ng-4000 ng/ml), C3 (88-171 mg/dl) and C4 (15-48 mg/dl) (McClendon Labs, UNC Healthcare, Chapel Hill, NC0. As noted the upper limit of detection for the MBL assay was 4000 ng/ml, thus those with levels above 4000 ng/ml were assigned a level of 4000 ng/ml.

### Statistics

Data are represented as mean ± standard deviation. Statistical analysis was performed using SigmaPlot, Systat Software (San Jose, CA). A two-tailed Student’s t test was used to compare MBL levels normally distributed between experimental groups. For data not normally distributed, non-parametric testing was performed using Mann–Whitney rank sum test.

## Results

**Figure 1:**
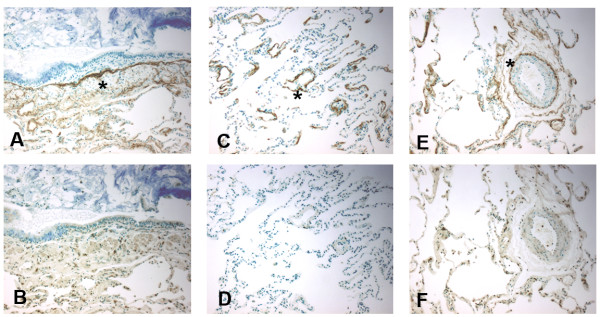
**Immunohistochemistry for mannose binding lectin (MBL) in lung allograft tissue (10X) during cold ischemia as detected by anti-MBL antibody (mouse anti-human), DAB, and methylene green counterstain.** MBL binds to airway basement membrane (star), **A**, alveolar capillary junctions (star), **C**, and endothelium (star) **E**. Isotype controls (mouse IgG1, κ) shown in **B**, **D**, and **F**, respectively.)

**Figure 2:**
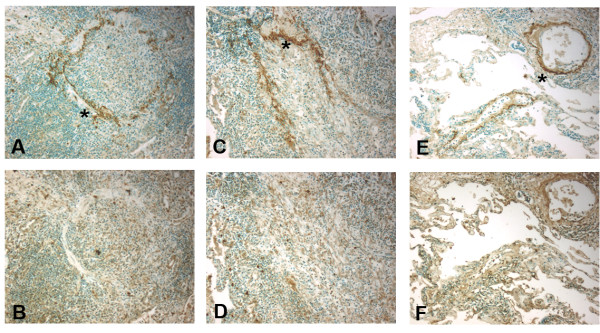
**Immunohistochemistry for mannose binding lectin (MBL) in lung allograft tissue (10X) in explanted lung allograft tissue with bronchiolitis obliterans as detected by anti-MBL antibody (mouse-anti-human), DAB, and methylene green counterstain.** MBL binds to remnant airway basement membrane(star), **A** and **C**, and vasculature (star), **E**. Isotype controls (mouse IgG1, κ) shown in **B**, **D**, and **F**, respectively.

**Figure 3:**
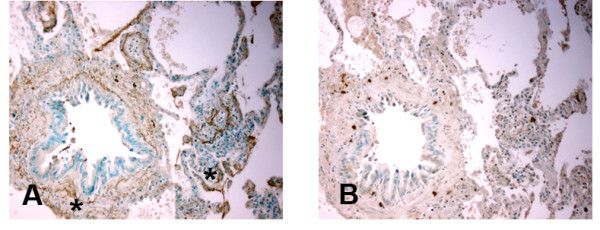
**Immunohistochemistry for mannose binding lectin (MBL) in lung allograft tissue (10X) in lung biopsies with non-transplant diffuse alveolar damage as detected by anti-MBL antibody (mouse-anti-human), DAB, and methylene green counterstain.** MBL binds to airway basement membrane and alveolar spaces (star), **A**. Isotype control (mouse IgG1, κ) shown in **B**.

**Figure 4:**
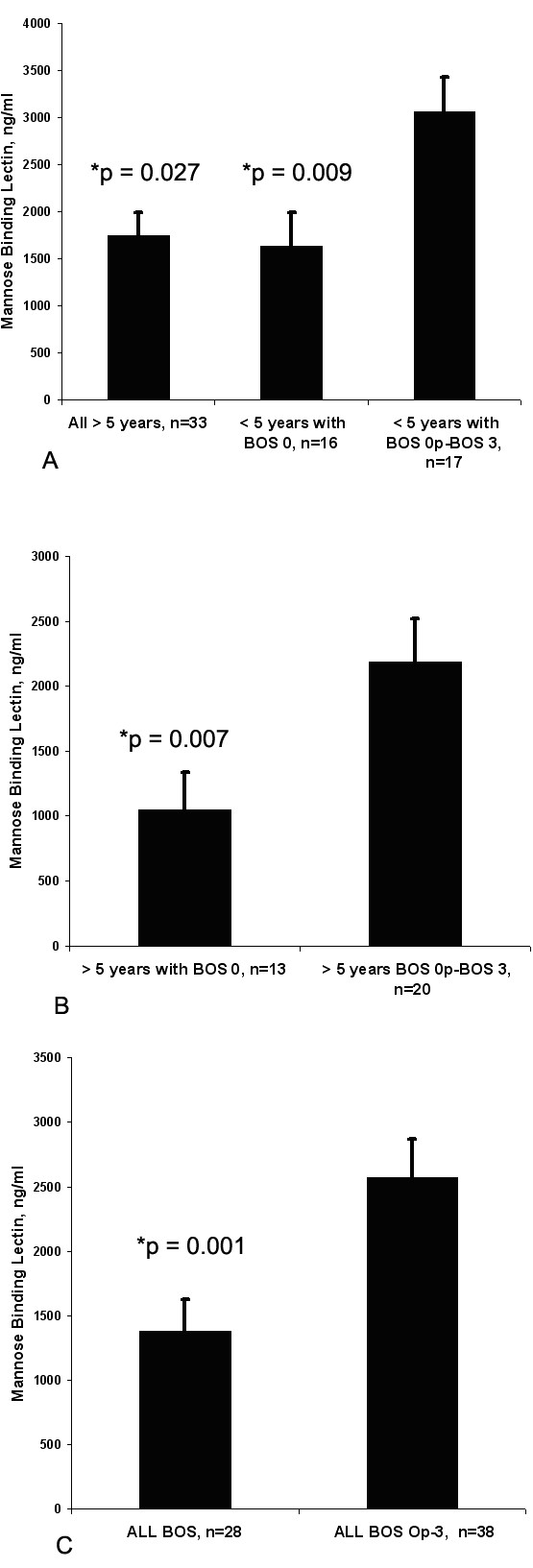
**Mannose binding lectin levels in the peripheral blood of lung transplant patients transplanted > 5 years or < 5 years, with BOS 0 compared to BOS Op-3.**Long-term > 5 years post-lung transplant patients (n = 33) had significantly lower levels of MBL in the blood compared to lung transplant patients < 5 years with BOS Op-3 (n = 10), 1738 ng/ml vs 3198 ng/ml, p = 0.027, **A**. Within the long-term > 5 years post-transplant cohort, patients with BOS 0 (n = 12) had significantly lower levels of MBL compared to those with BOS Op-3 (n = 21), MBL level 1,048 ng/ml vs 2186 ng/ml, p =0.007, **B**. Comparing all patients with BOS 0 (n = 29) vs. BOS Op-3 (n = 36), BOS 0 patients had significantly lower MBL and C3, 1378 ng/ml vs. 2578 ng/ml, p = 0.001, **C**.

**Figure 5:**
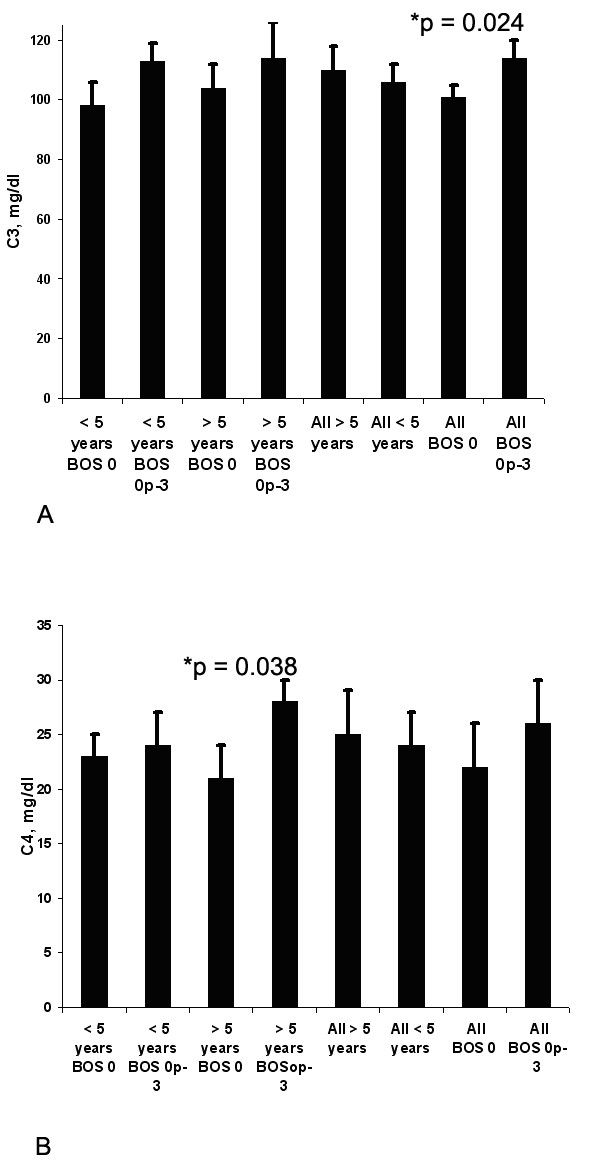
**Comparing all patients with BOS 0 (n = 29) vs. BOS Op-3 (n = 36), BOS 0 patients had significantly lower C3, 104 mg/ml vs. 114 mg/ml, p = 0.024, respectively, A.** Within the long-term survivors cohort, patients with BOS 0 (n = 12) had significantly lower levels of MBL and C4 compared to those with BOS Op-3, C4 level 21.6 mg/ml vs 28.7 mg/ml, p = 0.038, **B.**

1. Immunohistochemistry for MBL at the time of lung allograft implantation, from explanted lung allografts with BOS, and non-transplanted lung tissue with diffuse alveolar damage. Immunohistochemistry for MBL was performed on sections of lung allograft tissue from patients at the time of lung allograft implantation paralleling cold ischemia, (n = 6) (Figure 1), explanted lung tissue from patients retransplanted for bronchiolitis obliterans syndrome (BOS, n = 8) (Figure 2) and non-transplant patients with diffuse alveolar damage (DAD, n = 6) (Figure 3), in accordance with IRB protocols. MBL deposition was detected in all cold ischemia samples, localizing to vasculature (80.1 ± 12%), bronchial epithelial basement membranes (72.6 ± 11%), and along alveolar/capillary membrane (33.4 ±6%). BOS specimens demonstrated MBL along vasculature (88.5 ±8%), remnant bronchiolar basement membranes (52.3 ±15%), and alveolar/capillary basement membranes (18.4 ±6%). DAD tissue exhibited fainter deposition along vascular structures (27.7 ±11%) and bronchiolar basement membranes (54.3 ± 8%). Airway epithelial cells did not stain positive in any tissue specimens. These data demonstrate that MBL is widely distributed in allografted lung tissue at the time of transplantation as well as in chronic lung allograft rejection, localizing to basement membrane structures. Ongoing activation of the lectin complement pathway may support innate immune responses and complement binding, contributing to graft injury.Figure 1Immunohistochemistry for mannose binding lectin (MBL) in lung allograft tissue (10X) during cold ischemia as detected by anti-MBL antibody (mouse anti-human), DAB, and methylene green counterstain.Immunohistochemistry for mannose binding lectin (MBL) in lung allograft tissue (10X) during cold ischemia as detected by anti-MBL antibody (mouse anti-human), DAB, and methylene green counterstain. MBL binds to airway basement membrane (star), A, alveolar capillary junctions (star), C, and endothelium (star) E. Isotype controls (mouse IgG1, κ) shown in B, D, and F, respectively.)Figure 2Immunohistochemistry for mannose binding lectin (MBL) in lung allograft tissue (10X) in explanted lung allograft tissue with bronchiolitis obliterans as detected by anti-MBL antibody (mouse-anti-human), DAB, and methylene green counterstain.Immunohistochemistry for mannose binding lectin (MBL) in lung allograft tissue (10X) in explanted lung allograft tissue with bronchiolitis obliterans as detected by anti-MBL antibody (mouse-anti-human), DAB, and methylene green counterstain. MBL binds to remnant airway basement membrane(star), A and C, and vasculature (star), E. Isotype controls (mouse IgG1, κ) shown in B, D, and F, respectively.Figure 3Immunohistochemistry for mannose binding lectin (MBL) in lung allograft tissue (10X) in lung biopsies with non-transplant diffuse alveolar damage as detected by anti-MBL antibody (mouse-anti-human), DAB, and methylene green counterstain.Immunohistochemistry for mannose binding lectin (MBL) in lung allograft tissue (10X) in lung biopsies with non-transplant diffuse alveolar damage as detected by anti-MBL antibody (mouse-anti-human), DAB, and methylene green counterstain. MBL binds to airway basement membrane and alveolar spaces (star), A. Isotype control (mouse IgG1, κ) shown in B.

2. MBL levels in the peripheral blood after lung transplantation. A. Long-term > 5 years post-lung transplant (n = 33) patients, irregardless of BOS stage, had significantly lower levels of MBL in the blood compared to lung transplant patients < 5 years with BOS Op-3 (n = 10), 1738 ± 250 ng/ml vs 3198 ± 370 ng/ml, p = 0.027, and similar levels to lung transplant patients < 5 years with BOS 0 (n = 12), 1738 ± 250 ng/ml vs 1808 ± 345 ng/ml. Similarly, patients with BOS 0 < 5 years post-lung transplant had significantly lower levels of MBL compared to lung transplant patients < 5 years with BOS Op-3, 1808 ± 345 ng/ml vs. 3198 ± 370 ng/ml, p = 0.009 (Figure 4A). Approximately 4/33 (12%) of patients > 5 years post-transplant were MBL deficient (< 5 ng/ml), compared to 0/33 of patients < 5 years post-transplant (p = 0.057).Figure 4Mannose binding lectin levels in the peripheral blood of lung transplant patients transplanted > 5 years or < 5 years, with BOS 0 compared to BOS Op-3.Mannose binding lectin levels in the peripheral blood of lung transplant patients transplanted > 5 years or < 5 years, with BOS 0 compared to BOS Op-3. Long-term > 5 years post-lung transplant patients (n = 33) had significantly lower levels of MBL in the blood compared to lung transplant patients < 5 years with BOS Op-3 (n = 10), 1738 ng/ml vs 3198 ng/ml, p = 0.027, A. Within the long-term > 5 years post-transplant cohort, patients with BOS 0 (n = 12) had significantly lower levels of MBL compared to those with BOS Op-3 (n = 21), MBL level 1,048 ng/ml vs 2186 ng/ml, p =0.007, B. Comparing all patients with BOS 0 (n = 29) vs. BOS Op-3 (n = 36), BOS 0 patients had significantly lower MBL and C3, 1378 ng/ml vs. 2578 ng/ml, p = 0.001, C. B. Within the long-term > 5 years post-transplant group, patients with BOS 0 (n = 12) had significantly lower levels of MBL compared to those with BOS Op-3 (n = 21), MBL level 1,048 ± 288 ng/ml vs 2186 ± 333 ng/ml, p =0.007, (Figure 4B). C. Comparing all patients with BOS 0 (n = 30) vs. BOS Op-3 (n = 36), BOS 0 patients had significantly lower MBL, 1378 ± 275 ng/ml vs. 2578 ± 390 ng/ml, p = 0.001, (Figure 4C).

3. Assessment of MBL levels compared to time post-transplantation. MBL levels were compared to time post-transplantation by linear regression in order to determine if there was a significant effect of time post-transplant. All 66 MBL levels obtained were analyzed compared to a range of 2–22 years post-transplant. The resulting values were R = 0.31, R2 = 0.10, and p = 0.009, suggesting that MBL levels do decrease over time but that the correlation is fair at best.

4. Complement levels in the peripheral blood after lung transplantation. Comparing all patients with BOS 0 (n = 30) vs. BOS Op-3 (n = 36), BOS 0 patients showed significantly lower C3, 101 ± 19.8 mg/ml vs. 114 ± 25.2 mg/ml, p = 0.024, respectively, (Figure 5A). There were also trends toward lower C3 levels in the cohorts divided by transplant time (< 5 vs > 5 years). Within the long-term > 5 years post-transplant group, patients with BOS 0 (n = 12) had significantly lower levels of C4 compared to those with BOS Op-3 (n = 21), C4 level 21.6 ± 5.2 mg/ml vs 28.7 ± 6.3 mg/ml, p = 0.038 (Figure 5B). There were also trends toward lower C4 levels in BOS 0 vs BOS p-3 in those <5 yrs from transplant.Figure 5Comparing all patients with BOS 0 (n = 29) vs. BOS Op-3 (n = 36), BOS 0 patients had significantly lower C3, 104 mg/ml vs. 114 mg/ml, p = 0.024, respectively,Comparing all patients with BOS 0 (n = 29) vs. BOS Op-3 (n = 36), BOS 0 patients had significantly lower C3, 104 mg/ml vs. 114 mg/ml, p = 0.024, respectively, A. Within the long-term survivors cohort, patients with BOS 0 (n = 12) had significantly lower levels of MBL and C4 compared to those with BOS Op-3, C4 level 21.6 mg/ml vs 28.7 mg/ml, p = 0.038, B.

## Discussion

Innate immunity, encompassing anti-microbial responses, Toll-like receptors, complement, interleukin-1, macrophages, neutrophils, dendritic cells, and NK cells, is an area of intense investigation in solid organ transplantation. Mannose binding lectin (MBL) participates in the lectin pathway of complement activation and innate immunity, binds to apoptotic cells and microbes, and modulates inflammation. Although protective in enhancing responses to microbes, we hypothesize that elevated levels of MBL may contribute to allograft injury and bronchiolitis obliterans syndrome (BOS) after lung transplantation. Therefore MBL levels in patients > 5 years post-transplant and patients with BOS 0 will be reduced compared to patients transplanted < 5 years and patients with BOS Op-3.

Firstly, we have identified MBL binding to graft endothelial surfaces and airway basement membranes during ischemia-reperfusion and bronchiolitis obliterans, demonstrating the presence of MBL during initial implantation and during chronic alloimmune injury. Secondly, we have determined that patients > 5 years post-transplant had significantly lower levels of MBL compared to those less than 5 years post-lung transplantation. Thirdly, within each group, those with BOS 0 had significantly lower MBL levels compared to those with BOS Op-BOS 3. Fourthly, C3 complement levels were significantly lower in BOS 0 patients compared to those with BOS O –Op, and C4 complement levels were significantly lower in BOS 0 patients who were > 5 years post-transplant. These data further the evidence for innate immune activation coexisting with adaptive immune responses, and suggest that components of the complement system may impact upon long-term graft survival and bronchiolitis obliterans syndrome. Furthermore, in the > 5 year post-transplant group, 12% had levels below detection. This constitutes a significantly greater than the estimated prevalence of functional MBL deficiency than in the general population ~ 5%.

Mannose binding lectin, as a member of the collectin family, participates in host immunity and opsonization of bacteria, playing a role in autoimmunity, cardiovascular disease, and sepsis. As a member of the collectin family involved in host defense, MBL deposition may be expected in the lung during injury or inflammation, however, it has not been demonstrated in lung tissue sections previously. Evidence from experimental studies suggests that alloantibody binding to endothelial cells promotes complement activation, with subsequent binding of MBL to carbohydrates on the Fc portion of the antibody [38]. Members of the collectin family including MBL and H-ficolin have been associated with antibody-mediated rejection and ischemia-reperfusion in clinical studies and experimental models outside the arena of lung transplant. In renal transplant patients, MBL was present during ischemia-reperfusion and delayed renal graft function [39,40], and H-ficolin present during routine surveillance kidney biopsies [41]. In experimental models, MBL deposited during renal ischemia-reperfusion was inhibited by C1q inhibitor infusion [39] which achieved graft protection. In a cardiac model of antibody-mediated rejection, MBL contributed to C4d deposition, as in the absence of MBL, complement and non-complement fixing antibodies were unable to cause C4d deposition [42]. Our finding of MBL deposition in ischemia-reperfusion, diffuse alveolar damage, and bronchiolitis obliterans are in keeping with these studies.

In contrasting patients > 5 years post-transplant and recently transplanted patients, lower MBL levels trended down the longer time post-transplant, however, MBL level and time post-transplant were only fairly correlated per linear regression analysis. In addition, those with BOS 0 had the lowest levels of MBL within each of the groups compared to those with BOS Op −3. Studies of the role of MBL in solid organ transplantation have identified unique features associated with differing MBL levels and genotypes. Low levels of MBL were associated with a higher risk of infection after liver transplantation [43]. However, low levels of MBL conferred relative protection from alloimmune injury. MBL deficiency was also associated with fewer episodes of acute graft rejection after cardiac transplantation [44]. Similarly, low serum MBL proved protective in kidney and kidney-pancreas transplantation [33,34]. Studies addressing MBL and lung transplantation found that donor X-allele and LXPA haplotype, associated with low serum MBL production, conferred allograft protection from BOS [45], and similar to our findings, that high levels of MBL were associated with poorer outcomes after lung transplantation [36]. Lastly, our survey of C3 and C4 levels demonstrated significantly lower C3 levels associated with BOS 0, and long-term survivors with BOS 0 had the lowest C4 levels of all groups. Although complement levels deplete during acute exacerbations of autoimmune and rheumatological diseases, a broad variety of chronic inflammatory disease states including myocardial inflammation, pancreatic carcinoma, and age-related macular degeneration are associated with higher levels of complement [46-50]. Similarly, our findings suggest that higher complement levels present in higher stages of BOS may coincide with chronic inflammation.

There were several limitations to this study. Regarding the immunohistochemistry, the number of samples was limited, and analysis of normal lung tissue was limited by lack of availability from surgical biopsies often performed for diagnosis of lung disease. Transbronchial biopsies would be an alternative, but given the low yield for detecting BOS, was not one of the study’s initial objectives. Nevertheless, transbronchial biopsies would be useful to show the absence of MBL in those patients without BOS. The small numbers of patients in each group may warrant a larger analysis to fully confirm the findings, and the majority of patients transplanted harbored cystic fibrosis. In this regard, these data including mostly CF patients amplifies the complex role of MBL in biology and disease. MBL-2 acts as a gene modifier for CF disease and while MBL deficiency portends worse CF disease pre-transplant [51], likely related to host defense, low levels post-transplant are protective, highlighting the benefit of reduced innate immunity. We measured MBL levels after transplantation on the assumption that MBL is genetically determined and that levels do not vary significantly, although MBL levels may vary within a defined range during infection and inflammation as an acute phase reactant. However, serial MBL levels in a renal transplant group did not vary after transplant, as confirmed by Ibernon and colleagues [52]. We did not analyze structural gene mutations of MBL nor polymorphisms, which are associated with differential expression of MBL, for example, to see if the MBL deficient patients had the LXP promoter haplotype, which is associated with low serum levels. Certainly haplotype identification would further define low MBL producers with a genetic susceptibility and possibly those less likely to develop BOS after transplant. Nevertheless, in other studies MBL levels alone were sufficient to predict graft dysfunction after renal transplant [33,34]. Lastly, low levels of MBL may confer improved survival remains to be elucidated. While MBL may bind to antibody Fc portions adherent to basement membrane and endothelial cell surfaces, MBL may also be involved in opsonization, phagocytosis, and apoptosis [53]. The latter set of functions would be carried out by macrophages through calreticulin. MBL binds calreticulin [54] which plays a role in antigen presentation [55] and serves as a trigger for innate immune responses in autoimmunity [56], DC maturation, and T cell responses [57]. Conceivably, less functional uptake of antigen and apoptotic particles may lessen alloimmune stimulus. Future studies may better address the mechanism of MBL related graft injury, as part of antibody-mediated rejection or alteration of antigen-presentation through impaired apoptosis.

In conclusion, MBL may contribute to graft inflammation and rejection, serving to prime adaptive immunity during ischemia-reperfusion, recurrent acute lung allograft rejection, and bronchiolitis obliterans. If mannose binding lectin is an active participant in lung allograft injury at the initiation of ischemia reperfusion through the continuum of acute and chronic lung allograft rejection, current practice would be altered towards complement inhibition and improved methods for detection of humoral rejection. Our data suggests that MBL is deposited within critical vascular endothelium, epithelial basement membrane structures, colocalizing at sites which succumb to long-term fibrosis. Moreover, elevated MBL in the peripheral blood of patients with early to late stages of BOS may prove to be a biomarker for an increased risk of chronic graft injury. Reduction of MBL levels and the elucidation of MBL ligands expressed by the lung allograft may provide targets for therapeutic inhibition.

## Abbreviations

BOS, Bronchiolitis obliterans syndrome; MNL, Mannose binding lectin.

## Competing interests

The authors declare that they have no completing interests.

## Authors’ contributions

SJB participated in performance of research, RMA participated in the writing of the paper and data analysis, AAM participated in performance of the research, SLT participated in the writing of the paper, IPN participated in research design, writing of the paper, and data analysis. Support for this study granted by the American Society of Transplantation Clinical Science Faculty Development Grant. All authors read and approved the final manuscript.
